# Changes in self-rated health, quality of life, and psychological flexibility among adults with overweight over a 24-month acceptance and commitment therapy–based lifestyle intervention

**DOI:** 10.1177/20551029241302977

**Published:** 2024-11-21

**Authors:** Mari Petäjä, Raimo Lappalainen, Tarja Kettunen, Päivi Lappalainen, Kirsikka Kaipainen, Joona Muotka, Kirsti Kasila

**Affiliations:** 1 4167 Jamk University of Applied Sciences, Finland; 2 4168 University of Jyväskylä, Department of Psychology, Finland; 3 541605 University of Jyväskylä Faculty of Sports and Health Sciences, Finland; 4 549813 Tampere University Faculty of Information Technology and Communication Sciences, Finland

**Keywords:** acceptance and commitment therapy, health-related quality of life, lifestyle change, overweight, psychological flexibility, self-rated health

## Abstract

This study investigated the impact of online psychological flexibility training and support provided by peers on self-rated health (SRH) and health-related quality of life (HRQoL) among adults with overweight. It was a secondary analysis of a single-arm multi-center intervention study that was conducted in a real-life context. In a 2-years acceptance and commitment therapy (ACT) lifestyle intervention, participants (*N* = 177) engaged in online ACT activities while receiving support from trained peers. Participants completed surveys at baseline, 6, 12, and 24 months. The research data were analyzed with structural equation modelling. At baseline, the participants with high SRH (*n* = 83) had higher psychological flexibility and HRQoL than did participants with low SRH (*n* = 94). At 24 months, the participants with low SRH at baseline reported increases in SRH, vitality and depression dimensions of HRQoL, and psychological flexibility. Increased psychological flexibility was associated with improved SRH.

## Introduction

Non-communicable diseases (NCD) currently induce 74% of all deaths each year. These include diagnoses such as cardiovascular diseases, cancer, chronic respiratory distress and diabetes, which are frequently caused by overweight and obesity (WHO, 2022a). In a recent report, in the United States the increasing prevalence of adults with obesity is 42.5%, and with overweight 31.1% (Fryar et al., 2020). In Europe, the proportion of adults living with overweight or obesity is presently 59% (WHO, 2022b).

Lifestyle interventions are typically implemented for individuals with an NCD or at risk of one, as well as for those who intend to change their own nutritional and physical exercise habits along with increasing their broader well-being. In addition to possible changes achieved in physical health and health behavior, the achieved changes within lifestyle interventions may also affect a person’s perception of their overall well-being and health. In this study, overall well-being is examined from three perspectives: psychological flexibility, self-rated health, and health-related quality of life.

In recent years, a growing number of lifestyle interventions concentrating on physical health have applied an overall well-being approach. First, studies have shown that increased psychological flexibility has acted as a moderator in lifestyle changes (Forman and Butryn, 2015; Ivanova et al., 2016; Punna et al., 2021; Yildiz, 2020). Psychological flexibility comprises a person’s processes of how to adapt in fluctuating situations, recreate mental capacity, shift perspectives when needed, and adjust oneself to challenging desires, needs, and disciplines (Hayes et al., 2006; Kashdan and Rottenberg, 2010). With lifestyle changes, those skills and attitudes often play a critical role in a person’s change process (Forman and Butryn, 2015). Psychological flexibility can be trained by utilizing acceptance and commitment therapy (ACT), which aims at helping people to take value-based actions in a variety of life situations that involve a range of feelings and emotions. ACT uses acceptance, mindfulness, value processes, and committed action as methods to increase one’s psychological flexibility (Hayes et al., 2006; Lillis and Kendra, 2014). ACT has also been shown to be effective in online self-help interventions for mental well-being (Klimczak et al., 2023).

In addition to changes in psychological flexibility and daily health-related habits, psychological flexibility training during an ACT-based intervention may have an impact on multiple aspects of individual well-being. Psychological flexibility encompasses the ability to adapt to changing situations and adjust to challenging desires as well as experiential avoidance. Moreover, those are elements that may generate positive experiences for an individual with their well-being over the long term (Lillis and Kendra, 2014; Palmeira et al., 2017). In a recent paper, Villanueva et al. (2023) demonstrated that, as a moderator, high psychological flexibility was commonly associated with the experience of better moods despite the strain of an upsetting situation or environment. Within the current lifestyle intervention, ACT has been established as a framework because the training of psychological flexibility has been shown to have potential to increase individuals’ flexible thinking and acting as well as their ability to perceive the obstacles and challenges they face in changing their habits (Yildiz, 2020). Compared to other third-wave CBT approaches, ACT was chosen because it has been widely investigated in more than 1000 RCTs. Furthermore, meta-analyses have demonstrated the positive effects of ACT on healthy behaviors, psychological symptoms, and quality of life under a broad range of conditions, such as health problems, pain, and mental disorders (Gloster et al., 2020), as well as with cardiovascular diseases (Zhang et al., 2023a). Additionally, the current study is based on the extensive experience of the research group with ACT-based studies.

Second, a relevant perspective on overall well-being is self-rated health (SRH). This is based on the individual’s perception of their state of health rated on a single five-point scale (Idler et al., 2000; Jylhä, 2009). SRH has been shown to predict an individual’s health in the future (Heistaro et al., 2001), and the poor SRH is associated with an increased risk of NCDs such as cardiovascular events and coronary heart disease (May et al., 2006). Regarding to lifestyle behavior, a relationship between poor SRH and obesity has been identified (Raghupathi and Raghupathi, 2018). Additionally, the relation between poor SRH and increased risk of mortality is strong (DeSalvo et al., 2006; Heistaro et al., 2001; Idler et al., 1990) among both women and men, and it is only partially explained by medical history, cardiovascular risk factors such as low physical activity, and education (Heistaro et al., 2001). However, SRH is a reliable measure of health risk assessment that can provide information on the individual level about the need for counselling on health behavior change and clinical trials (DeSalvo et al., 2006; Jylhä, 2009).

People’s concerns about their personal state of health and well-being, including attempts to and success in increasing healthy actions, may reflect how people experience their quality of life (Dochat et al., 2021). The World Health Organization has defined quality of life as “individuals’ perceptions of their position in life in the context of the culture and value systems where they lived and in relation to their goals, expectations, standards and concerns” (WHO, 1996: 354). More specifically, and as a third perspective on overall well-being, health-related quality of life (HRQoL) is an indicator of the state of well-being an individual experiences. In addition, it can be considered a long-term endpoint and outcome for lifestyle changes, as it indicates the favorable consequences of those changes (Haraldstad et al., 2019). With NCDs such as diabetes type 2, there is an increased risk of distress, thus improving quality of life should be targeted within lifestyle interventions (Laverty et al., 2023; Onu et al., 2022).

Relatively little is known, or the evidence remains contradictory, about the relations between the perspectives on overall well-being such as psychological flexibility, self-rated health and quality of life in the context of overweight and long-term lifestyle changes. However, recent reviews have shown that online ACT interventions have been effective in enhancing quality of life (Han et al., 2022) for individuals living with chronic conditions as well (Herbert et al., 2022). Additionally, training in ACT has improved quality of life among adults with chronic pain (Ma et al., 2023) and cancer patients (Zhang et al., 2023b).

Furthermore, Chew et al. (2023) found, in their review of the effects of ACT interventions for adults with obesity or overweight, favorable changes in psychological flexibility but not in quality of life. Earlier, an RCT study showed that ACT training led to better self-rated health among stroke survivors, but the same study reported no changes in quality of life (Majumdar and Morris, 2019). Moreover, Lillis et al. (2009) found that a short ACT weight loss intervention led to improved psychological flexibility, which also acted as a mediator for improved quality of life. In Forman et al. (2009), a 12-weeks ACT intervention for weight loss yielded a higher quality of life. This result, along with the weight loss, was maintained at a 6-months follow-up point. In a 10-weeks ACT intervention for overweight women, Palmeira et al. (2017) showed that mindfulness training increased physical activity and quality of life.

However, evidence is lacking regarding how lifestyle interventions influence overall well-being among overweight adults over a longer period. In a previous study, we found that participants in an ACT-based lifestyle intervention improved their psychological flexibility and that those with low physical activity increased their activity (Punna et al., 2021). In this study, we were interested in increasing our understanding of the overall long-term well-being changes among adults with overweight by investigating the effects of a 24-months ACT-based lifestyle intervention on their self-rated health, various dimensions of quality of life, and psychological flexibility. To examine the changes in self-rated health, we separately emphasized participants with high SRH and low SRH at baseline. The research questions were as follows:

1. What are the characteristics of the participants with high and low self-rated health at baseline, and how do these characteristics differ?
2. How does ACT training impact self-rated health, the dimensions of health-related quality of life and psychological flexibility among participants with high and low self-rated health?
3. Is increased psychological flexibility associated with self-rated health and the dimensions of health-related quality of life among all the participants and with those who reported low self-rated health at baseline?

## Methods

### Data collection and intervention

The research data have been collected between 2015 and 2018 as a part of *The Journey of Change* -project in Central Finland, which was undertaken within municipalities’ public health services in a real-life context. The current intervention study as well as its specified intervention protocol, the procedure of the data collection, and the measurement instruments were approved by participating public health centers and by the Ethics Committee of the Central Finland Health Care District. Health professionals recruited volunteers to an overall well-being–oriented, peer-tutored lifestyle change group. The inclusion criteria for participants were as follows: (1) age 18 or older, (2) BMI of 25 or higher, (3) a verbally expressed willingness to make lifestyle changes, (4) being capable of written activities, and (5) having access to the Internet. At the first group meeting, participants were given written and oral information about the study and the intervention, and gave their written consent to participate in the study as volunteers. Participants (*N* = 177) were allocated into 16 intervention groups, with 7 to 17 individuals in each group at the beginning of the intervention. The dropout rate over the 24-months intervention was 40.7% (*n* = 105 at 24 months).

The intervention was composed of two periods: a supported period from 0 to 12 months, and a self-directed period from 12 to 24 months (Supplement 1: Figure S1). The supported period consisted of four group meetings tutored by a group leader, a health professional and a peer tutor; two 6-weeks ACT-based online modules followed by feedback from a peer tutor; and two phone calls by the peer tutor. The self-directed period consisted of one 6-weeks ACT-based online module without feedback, two phone calls and one face-to-face group meeting. In group meetings, participants’ ongoing lifestyle change processes were discussed and reflected on. They also shared reciprocal support to overcome the challenges and obstacles they encountered. The online modules provided a wide range of ACT-based activities to facilitate and help participants reflect on the feelings, thoughts, and emotions they were experiencing during their change process. These were discussed and shared in the group meetings, allowing for peer-reflections among participants and trained peers. The 10 trained peers, who all had personal experience with chronic conditions, had received 8 months of training (a total of 103 h of classroom lectures with additional homework) to serve as peer tutors in health and social services. In addition, they attended additional training focusing on peer tutoring in the ACT-based online lifestyle change intervention. See Punna et al. (2021) for a detailed description of the data collection, intervention and online modules.

### Measurement instruments

#### Demographic variables

The following demographic variables were collected at baseline: sex, age, working status, diagnosed long-term diseases, body weight (kg), and height (cm). The calculated *BMI* is a measure of an individual’s body weight in relation to their height. A score of 18 to 24.9 indicates normal weight, 25≤ overweight with health risks, and 30≤ obesity with severe risks for comorbidities (WHO, 2021).

#### Process variables

Psychological flexibility was examined using two measures. The Acceptance and Action Questionnaire (AAQ-II) provides a broad approach to psychological flexibility (Bond et al., 2011), and the White Bear Suppression Inventory (WBSI) explores thought suppression as a dimension of psychological inflexibility (Wegner and Zanakos, 1994). The AAQ-II has seven multiple-choice questions with the total score ranging from 7 to 49 where a lower score indicates higher psychological flexibility. The WBSI is a questionnaire consisting of 15 multiple choice questions to assess obsessional thinking with a depressive and anxious aspect. In WBSI, the lower scores mean less thought suppression. In this study, Cronbach’s alphas were .92 for AAQ-II and 0.93 for WBSI at baseline. See Punna et al. (2021) for a detailed description of the process variables.

#### Outcome variables

The main outcome measure was self-rated health (SRH). It was examined with a single question that was answered using 5-point scale. The question invited the individual to reflect on their health (Bond et al., 2006; DeSalvo et al., 2006; Idler et al., 1990; Mossey and Shapiro, 1982) by asking “Would you say your health is…?” with response options being *good* (1), *quite good* (2), *average* (3), *quite poor* (4), and *poor* (5). SRH has been shown to establish the respondent’s perspective effectively in multiple aspects, such as functional limitations and cognitive impairment (Bond et al., 2006). The measure shows a strong predictive power of individual health and functionality. It also encompasses a respondent’s perspectives on their physical state of health, medical diagnoses, prescribed medicines and treatments, as well as various bodily sensations and symptoms such as pain, fatigue and low mood (Benyamini, 2011; Jylhä, 2009).

Health-related quality of life (HRQoL) can be measured and investigated with both generic and disease-specific measures (Helmiö et al., 2011; Sintonen, 2001). In this study, due to heterogenic data population, HRQoL was measured with a generic, standardized, self-administered 15D questionnaire (Alanne et al., 2015; Sintonen, 2001). It consists of 15 dimensions of HRQoL: breathing, mental function, speech, vision, mobility, usual activities, vitality, hearing, eating, elimination, sleeping, distress, discomfort and symptoms, sexual activity, and depression. Respondents evaluate each dimension using a 5-point scale. For example, for vitality, the scale is as follows: (1) I feel healthy and energetic. (2) I feel slightly weary, tired or feeble. (3) I feel moderately weary, tired or feeble. (4) I feel very weary, tired and feeble, almost exhausted. (5) I feel extremely weary, tired or feeble, totally exhausted (Health, 2011). According to the three-stage valuation process of the 15D questionnaire, the level values are generated on a scale of 0–1, where 1 refers to full health with no problems and 0 indicates being dead (Helmiö et al., 2011). From a clinical perspective, the minimum change has been considered to be between ≥0.015 (Alanne et al., 2015) and ≥0.03 (Helmiö et al., 2011), so that an individual can notice the difference in HRQoL themselves. The 15D results can be used as a single score measure as well as a profile measure (Alanne et al., 2015). The 15D has been tested for its reliability and validity, and has been considered a sensitive measure for changes in HRQoL. In addition, the measure has a good discriminatory power (Sintonen, 2001). In this study, the alpha was .83 at baseline.

### Statistical analysis

Descriptive statistics were calculated to describe the sample characteristics. To address the first research question, the sample was divided into high SRH (including good and quite good) and low SRH (including average, quite poor and poor) groups. The differences between the two SRH groups at baseline were analyzed using SPSS 28 for continuous variables with an independent sample *t* test, and categorical variables with crosstabs and a chi-square test.

In the second research question, the aim was to explore possible changes in SRH, health-related quality of life with its dimensions, and psychological flexibility. The analysis was conducted in three stages. The first stage was with all the data, the second was with the participants who reported high SRH at baseline and the third was with those who showed low SRH at baseline. All the variables were analyzed as continuous. The changes, significances, effect sizes and correlations were analyzed with Mplus version 8.4 (Muthén and Muthén, 2017) using the full information maximum likelihood (FIML). The statistical analysis was conducted with the maximum likelihood robust (MLR) method, which is applicable to skew distributions. All the participants meeting the inclusion criteria (*N* = 177) and completing the baseline measurements were included in the analyses; hence, the missing data were assumed to be missing at random (MAR). Furthermore, the changes at four time points (at 0, 6, 12, 24 months) were investigated with structural equation modelling using latent change scores. The Wald test (W) was used for investigating whether mean change was significant at least between two time points, when a change has been measured using four time points. Additionally, the effect sizes (Cohen’s *d*) were calculated by mean difference between two measurement points, and they were divided by pooled standard deviation between measurements.

To address the third research question, correlations of the statistically significant changes over the intervention, meaning the change between 0 and 24 months, were investigated for all participants as well as for those with low SRH. In the correlation analysis, the changes in AAQ-II and WBSI between 0 and 24 months in the group with all participants were taken from the statistical analysis of our previous study (Punna et al., 2021), where it was found that psychological flexibility measured by AAQ-II and WBSI improved among all the participants (*N* = 177) over the 24-months intervention.

## Results

### Sample characteristics

All the participants were White living in Central Finland in an urban area or the countryside. Most of them were women (83.6%) and having obesity with a BMI of 30 ≤ (87.0%), and at least one long-term disease (85.9%) (Supplement 2: Table S1). The most prevalent self-reported long-term conditions at baseline were hypertension (41.5% of participants, *n* = 73), musculoskeletal disorder (39%, *n* = 69), diabetes (29.4%, *n* = 52), and allergy or asthma (27.7%, *n* = 49). Half of the participants were employed (49.2%) and the rest of them were retired, students, or on long-term family or sick leave.

### The characteristics of the participants with high and low self-rated health at baseline

Over half of the participants reported average or poor SRH (53%, *n* = 94). They showed lower scores in health-related quality of life and psychological flexibility as well as higher BMI than did participants with good SRH at baseline. Additionally, among participants with low SRH, the number of those reporting one or more long-term diseases was higher while the number of those reporting working status as employed was lower.

### Changes in self-rated health, health-related quality of life and psychological flexibility

Among all participants (*N* = 177), *self-rated health* improved over the 24-months intervention period, with the change being the largest over the first 12 months (see Table 1). Participants with low SRH at baseline reported significantly increased SRH at 12 months and the change was maintained at 24 months. Additionally, participants who reported high SRH at baseline also showed minor positive changes over the first year of the intervention.

**Table 1.** Changes in self-rated health (SRH) over the 24-months intervention on a scale from (1) good to (5) poor.

Variable	Mean	*p*	Variance	Cohen *d*
With all (*N* = 177) W(3), 13.044, *p* = .005				
Baseline	2.66	<.001	0.87	
Change 0**–**6 months	−0.11	.108	0.72	.12
**Change 6–12**	**−0.15**	**.040**	**0.61**	.15
Change 12–24	0.10	.225	0.70	−.10
**Change 0**–**12**	**−0.26**	**<.001**		.27
**Change 0**–**24**	**−0.16**	**.050**		.16
SRH high (*n* = 83) W(3), 8.883, *p* = .031				
Baseline	1.82	<.001	0.15	
**Change 0–6 months**	**0.23**	**.009**	**0.56**	**−.37**
**Change 6**–**12**	**−0.22**	**.019**	**0.53**	**.28**
Change 12–24	0.17	.188	0.80	−.19
Change 0–12	0.01	.916		−.02
Change 0–24	0.18	.132		−.25
SRH low (*n* = 94) W(3), 40.941, *p* <.001				
Baseline	3.39	<.001	0.35	
**Change 0**–**6 months**	**−0.42**	**<.001**	**0.66**	.56
Change 6–12	−0.11	.273	0.69	.12
Change 12–24	0.05	.595	0.60	−.06
**Change 0**–**12**	**−0.53**	**<.001**		**.67**
**Change 0**–**24**	**−0.47**	**<.001**		**.59**

W = Wald test.

Statistically significant changes are bolded in the table.

*The health-related quality of life* measured with 15D scores showed that most of the observed scores were positioned at the high end of the scale at baseline. The 15D scores did not change over the intervention, but certain dimensions of HRQoL did improve (Supplement 2: Table S2).

Among all the participants, the lowest scores in the HRQoL dimensions were in sleeping and discomfort and symptoms (Supplement 1: Figure S2). The sleeping dimension improved over the intervention period (change 0.041, *p* = .012) as did vitality (change 0.041, *p* = .006).

Among the participants with high SRH, the discomfort and symptoms dimension of HRQoL marginally decreased between 0 and 6 months (change −0.046, *p* = .013) which was the only statistically significant change within this group (Supplement 1: Figure S3, and Supplement 2: Table S2).

In contrast, within the group reporting low SRH at baseline, there were improvements in different dimensions of HRQoL (Supplement 1: Figure S4, and Supplement 2: Table S2). Vitality and depression especially improved over the first intervention year and, furthermore, the achieved changes were maintained throughout the second year. The changes in vitality over the intervention period (0.064, *p* = .001) and depression over 12 months (change 0.060, *p* = .002) were statistically significant.

Additionally, we noticed statistically significant changes in the vision dimension of HRQoL (coded as “SEE” in Supplement 1: Figures S2–S4) among all participants and those with low SRH. However, the finding is not relevant due the high baseline scores (All: mean 0.946, variance 0.017; SRH low: mean 0.920, variance 0.026) (Supplement 2: Table S2).

*Thought suppression* (WBSI) decreased significantly among both groups of SRH (SRH high: change −6.06, *p* < .001; SRH low: change −3.66, *p* = .004) (Supplement 2: Table S3). Among the SRH low group, *psychological flexibility* (AAQ-II) also increased over the first year of the intervention and afterwards over the 2-years intervention (change −2.34, *p* = .004).

### Associations between the observed changes

Among all participants over the entire 24-months intervention period, increased psychological flexibility (AAQ-II) was associated with improved self-rated health, while decreased thought suppression (WBSI), which indicates more psychological flexibility, was associated with improved self-rated health (Table 2). There was a correlation between SRH and vitality, which demonstrates that the improvement of self-rated health was related to the increase in the vitality dimension of HRQoL. Additionally, increased vitality was correlated with an increase in the sleeping dimension of HRQoL.

**Table 2.** Correlations (*p*) between statistically significant changes between 0 and 24 months.

All (*N* = 177)						
	AAQ-II	WBSI	SRH	Sleeping dimension of H RQoL	Vitality dimension of HRQoL	Vision dimension of HRQoL
AAQ-II		**.355 (.014)**	**.381 (<.001)**	−.199 (.085)	−.138 (.313)	.027 (.826)
WBSI	**.355 (.014)**		**.256 (.007)**	−.045 (.655)	−.103 (.249)	−.009 (.914)
SRH	**.381 (<.001)**	**.256 (.007)**		−.048 (.685)	**.234 (.014)**	−.034 (.679)
Sleeping dimension of HRQoL	−.199 (.085)	−.045 (.655)	−.048 (.685)		**.328 (.002)**	−.121 (.286)
Vitality dimension of HRQoL	−.138 (.313)	−.103 (.249)	**−.234 (.014)**	**.328 (.002)**		.006 (.951)
Vision dimension of HRQoL	.027 (.826)	−.009 (.914)	−.034 (.679)	−.121 (.286)	.006 (.951)	
SRH low (*n* = 94)						
	AAQ-II	WBSI	SRH	Depression dimension of HRQoL	Vitality dimension of HRQoL	Vision dimension of HRQoL
AAQ-II		**.421 (.002)**	**.231 (.029)**	**−.299 (.021)**	−.004 (.980)	.089 (.536)
WBSI	**.421 (.002)**		.221 (.068)	**−.308 (.007)**	−.120 (.904)	−.073 (.468)
SRH	**.231 (.029)**	.221 (.068)		**−.319 (.006)**	−.216 (.076)	.089 (.344)
Depression dimension of HRQoL	**−.299 (.021)**	**−.308 (.007)**	**−.319 (.006)**		.140 (.298)	−.037 (.834)
Vitality dimension of HRQoL	−.004 (.980)	−.015 (.904)	−.216 (.076)	.140 (.298)		−.073 (.578)
Vision dimension of HRQoL	.089 (.536)	−.073 (.468)	.089 (.344)	−.037 (.834)	−.073 (.578)	

Among participants with low SRH, increased psychological flexibility (AAQ-II) was associated with improvement in self-rated health and the depression dimension of HRQoL (Table 2). Decreased thought suppression (WBSI) as an element of psychological flexibility was also associated with improvements in the depression dimension of HRQoL. Furthermore, improved SRH and improved depression were correlated.

Among the group with high SRH, the only statistically significant change between 0 and 24 months was in thought suppression (WBSI).

## Discussion

### Interpretation of results

The current study investigated the effects of ACT training on self-rated health and health-related quality of life among participants with overweight over a 24-months lifestyle change intervention. It produced further evidence of the effects of peer-tutored ACT training on perspectives of overall well-being, such as self-rated health and the patterns of quality of life, over a long-term period. The findings showed that participants had varying levels of self-rated health at the start of the intervention. At the beginning of the intervention, participants reporting poor or average self-rated health demonstrated lower psychological flexibility and HRQoL and had a higher body mass index. They also more often reported a non-working status and had more long-term diseases than those reporting good self-rated health. Among all participants over the 24-months intervention period, self-rated health, and the vitality and sleeping dimensions of HRQoL improved. Furthermore, increased psychological flexibility was associated with increased self-rated health. Participants showing low self-rated health at the beginning of the intervention reported improvements in self-rated health, the vitality and depression dimensions of HRQoL, and psychological flexibility. The findings showed that their increased psychological flexibility was associated with increased self-rated health and an improvement in the depression dimension of HRQoL.

Based on the study findings, individuals with overweight reported diverse experiences of overall well-being. Individuals having low self-rated health also experienced lower psychological well-being and quality of life. However, a half of the study participants with overweight experienced good self-rated health and health-related quality of life as well as lower body mass index at the beginning of the intervention. The finding highlights that a person at health risk does not necessarily feel unhealthy or express poor well-being. It also clearly indicates that people at health risk should not be approached as a homogenous group, but rather as persons facing and living in individual health and well-being conditions.

The current study shows how psychological flexibility training may be a promising option for improving self-rated health. The finding can be emphasized since improved SRH, even without a measurable change in physical health, is meaningful and predictive concerning one’s health in the future (Benyamini, 2011). Previously, the significance of self-rated health (May et al., 2006; Raghupathi and Raghupathi, 2018) and quality of life (Laverty et al., 2023; Onu et al., 2022) among people living with non-communicable diseases have been strongly highlighted. Due to the poor self-rated health and health-related quality of life among people at health risk, with overweight or non-communicable diseases possibly indicating a higher risk of functionality and other health problems, we need to increase our understanding about methods for strengthening self-rated health and quality of life. Although there are different pathways to achieve the outcomes of lifestyle interventions, the current study demonstrates how the strengthening of psychological skills may generate improvements in overall well-being outcomes over the long term.

In this intervention study, the achieved changes occurred mostly during the first year of the ACT intervention, but they were also maintained during the second year. The results may reflect the setting of the program, where the first year was a supported period and the second year more a self-directed period carried out individually (Supplement 1: Figure S1). A positive aspect was that many of the achieved changes were also maintained during the second year. Furthermore, lifestyle change is a psychological process that focuses on an individual’s capability to see their own life and ability to maintain changes in daily health habits. In addition, training psychological flexibility is an individual process. It requires one’s own sense to reflect on daily life from different perspectives, such as what capacities, obstacles, strengths, and emotions one experiences. Taking small steps consisting of value-based actions can be challenging, as can applying ACT in one’s daily life. That is an aspect which justifies adding peer-tutoring as an intervention element in ACT training.

Several additional issues that can also be raised on the basis of the results. First, the use of WBSI to measure thought suppression as a dimension of psychological inflexibility has been demonstrated to be a reliable approach to assessing the incidence of unwanted thoughts. Furthermore, thought suppression also acts as a significant maintenance mechanism for a variety of clinical disorders (Schmidt et al., 2009). Due to its sensitivity as a measure, WBSI is applicable in assessing the change processes in a clinical setting (Schmidt et al., 2009). In this study, WBSI indicated a decrease in thought suppression in both the high and low SRH groups. Furthermore, the decrease in thought suppression was associated with self-rated health and the depression dimension of HRQoL, which possibly reflects the interactions between thought suppression and mental well-being. On the other hand, AAQ-II measuring a broader range of psychological flexibility improved only among participants with low self-rated health. Second, the improvement in the depression dimension of HRQoL suggested high associations with the improvements in psychological flexibility, changes in thought suppression as well as with self-rated health among participants with low self-rated health.

Our earlier study showed that participants with low participation in physical activity reported higher physical activity at the end of the ACT-based lifestyle intervention (Punna et al., 2021). Taken together, the findings from the two studies suggest that, an ACT-based peer-tutored lifestyle intervention can be beneficial to adults with low physical activity, low self-rated health and reporting low health-related quality of life in certain dimensions. While the current study supports previous findings that ACT seems to be a relevant approach to increasing psychological well-being in lifestyle interventions targeted at adults with health risks (Chew et al., 2023; Iturbe et al., 2022), it also highlights the importance of support in change. Aiming at lifestyle changes is a long-term process that benefits from evidence-based methods to facilitate that change, as well as from support that addresses participants’ individual challenges. The findings reinforce previous research about the effectiveness of peer-tutored interventions for people with NCD (Dale et al., 2012; Punna et al., 2019; Zhang et al., 2016), where social support provided by peers is considered advantageous in the change process. Those who benefitted the most from the intervention were participants whose SRH was initially average or poor.

### Study limitations and future research

The current study has a number of strengths, such as the 2-years study period. Achieving lasting lifestyle changes takes time and usually includes a variety of obstacles and successes experienced in individuals’ own everyday life context. Especially within self-assessed quality of life and health, the attained changes may be considered relatively stable over time. A long intervention period applied in a real-life context, enabled durable changes to become noticeable.

However, the study also has limitations that should also be taken into consideration when interpreting the results. First, the findings can explain the individual change processes on an average level, but not on an individual one. Even though the intervention was strongly influenced by the ACT model and took into account participants’ own needs and goals, the findings should be construed as reflecting phenomena more on an average level than those within individual processes (Hayes et al., 2019). However, behind the statistical findings there are a variety of unique change processes (Kasila et al., 2020). The results of the study emphasize the need for a more varied understanding of the connections between psychological flexibility, self-rated health, and the dimensions of quality of life.

Second, this was not a randomized controlled trial. We are not able to rule out the possibility that factors other than intervention-related ones, including life events such as moving to another region or changes in career or family relations, may have triggered the observed changes. Additionally, we chose not to use multiple testing when examining the correlations between the changes. Due to this, it is possible that some of the significant correlations may have developed at random. Therefore, the correlations with *p* -levels >.01 should be taken with caution. However, most of the significant correlations were <0.01.

The intervention was conducted as a part of public health services, and due to ethical issues, it was not possible to exclude any participants who met the inclusion criteria. However, a 2-years time perspective provided a longer period with which to follow the change processes, giving particular insight into the self-directed period of the ACT intervention, as well as identifying two SRH-groups, high and low, which made it possible to compare the processes of these participants. Third, the number of participants in the two SRH groups was relatively small. Fourth, men are underrepresented within these data, which should be noted when generalizing the results.

On the basis of our results, we call for further examination of individual differences in future studies, with a focus on health-related changes and quality of life (Hayes et al., 2019). In addition to psychological flexibility, there are other variables that may interact with self-rated health and quality of life. Kwan et al. (2023) suggested that self-continuity might be associated with better body satisfaction, thereby promoting stronger engagement in healthy behaviors. We also propose that future studies investigate self-rated health and quality of life outcomes, as well as their associations with other variables of well-being over the long-term.

However, more longitudinal research on lifestyle changes is called for due to the long-term nature of the change process. When applicable, a randomized controlled study would be able to assess the effectiveness of the interventions more precisely. Additionally, it is also necessary to understand more about the individual experiences of the change processes for health and quality of life, as well as about how enhanced psychological flexibility is manifested in the context of daily life.

## Conclusions

Individuals living with health risks experience the quality of their health and life in different ways. An intervention based on ACT and the promotion of psychological flexibility skills can impact overall well-being. This study provides novel evidence of the impact of peer-supported online acceptance and commitment therapy lifestyle intervention on psychological flexibility, self-rated health and quality of life. Lifestyle interventions aimed at increasing psychological flexibility have the potential to increase self-rated health, which is a strong predictor of functionality and health.

## Supplemental Material


Supplemental Material - Changes in self-rated health, quality of life, and psychological flexibility among adults with overweight over a 24-month acceptance and commitment therapy–based lifestyle intervention
Supplemental Material for Changes in self-rated health, quality of life, and psychological flexibility among adults with overweight over a 24-month acceptance and commitment therapy–based lifestyle intervention by Mari Petäjä, Raimo Lappalainen, Tarja Kettunen, Päivi Lappalainen, Kirsikka Kaipainen, Joona Muotka and Kirsti Kasila in Journal of Health Psychology Open.

Supplemental Material - Changes in self-rated health, quality of life, and psychological flexibility among adults with overweight over a 24-month acceptance and commitment therapy–based lifestyle intervention
Supplemental Material for Changes in self-rated health, quality of life, and psychological flexibility among adults with overweight over a 24-month acceptance and commitment therapy–based lifestyle intervention by Mari Petäjä, Raimo Lappalainen, Tarja Kettunen, Päivi Lappalainen, Kirsikka Kaipainen, Joona Muotka and Kirsti Kasila in Journal of Health Psychology Open.
