# Supplementary material for: Changes in self-rated health, quality of life, and psychological flexibility among adults with overweight over a 24-month acceptance and commitment therapy–based lifestyle intervention
Source: Health Psychol Open. 2024 Nov 21;11:20551029241302977. doi: 10.1177/20551029241302977 (PMC11583280; doi:10.1177/20551029241302977)
Supplement: Supplemental Material - Changes in self-rated health, quality of life, and psychological flexibility among adults with overweight over a 24-month acceptance and commitment therapy–based lifestyle intervention [file sj-pptx-1-hpo-10.1177_20551029241302977.pptx]

## Slide 1
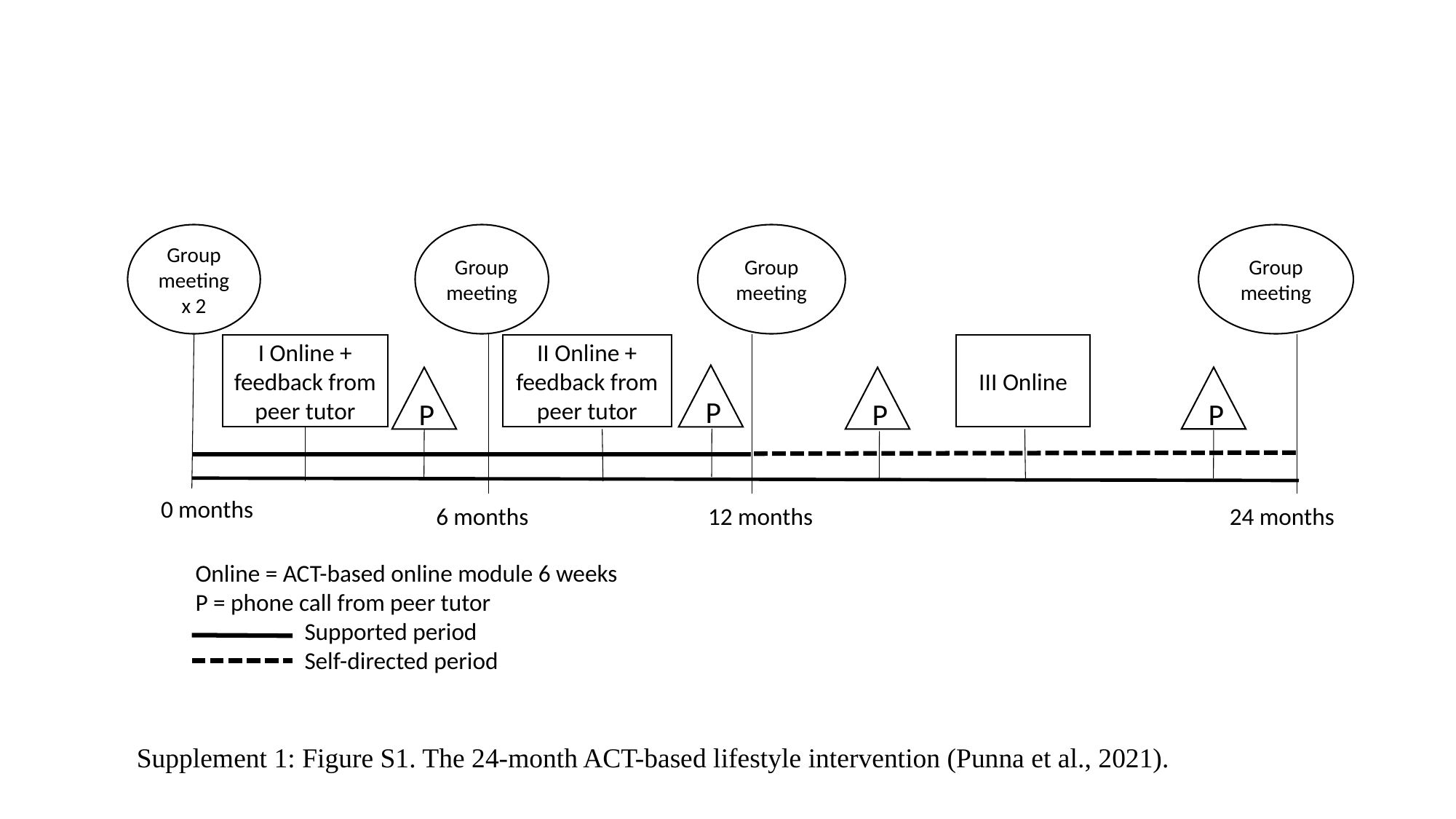

Group meeting x 2
Group meeting
Group meeting
Group meeting
I Online + feedback from peer tutor
II Online + feedback from peer tutor
III Online
P
P
P
P
0 months
6 months
12 months
24 months
Online = ACT-based online module 6 weeks
P = phone call from peer tutor
	Supported period
	Self-directed period
Supplement 1: Figure S1. The 24-month ACT-based lifestyle intervention (Punna et al., 2021).

## Slide 2
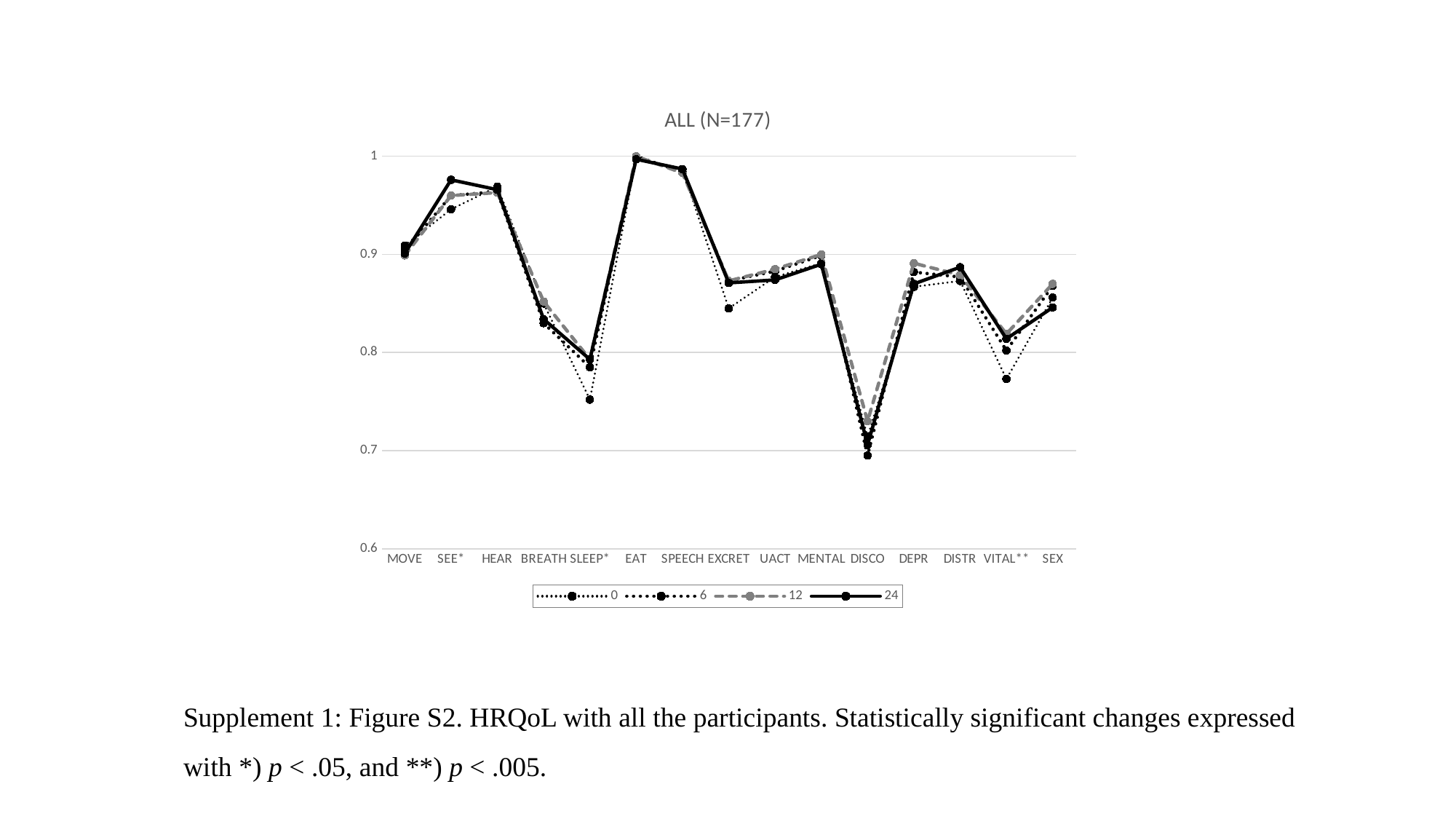

### Chart: ALL (N=177)
| Category | 0 | 6 | 12 | 24 |
|---|---|---|---|---|
| MOVE | 0.909 | 0.904 | 0.899 | 0.901 |
| SEE* | 0.946 | 0.96 | 0.96 | 0.976 |
| HEAR | 0.969 | 0.964 | 0.963 | 0.966 |
| BREATH | 0.85 | 0.83 | 0.852 | 0.834 |
| SLEEP* | 0.752 | 0.785 | 0.793 | 0.793 |
| EAT | 0.998 | 1.0 | 1.0 | 0.997 |
| SPEECH | 0.987 | 0.984 | 0.983 | 0.987 |
| EXCRET | 0.845 | 0.873 | 0.873 | 0.871 |
| UACT | 0.877 | 0.883 | 0.885 | 0.874 |
| MENTAL | 0.891 | 0.899 | 0.9 | 0.89 |
| DISCO | 0.714 | 0.695 | 0.73 | 0.706 |
| DEPR | 0.867 | 0.882 | 0.891 | 0.87 |
| DISTR | 0.873 | 0.877 | 0.879 | 0.887 |
| VITAL** | 0.773 | 0.802 | 0.819 | 0.814 |
| SEX | 0.856 | 0.868 | 0.87 | 0.846 |Supplement 1: Figure S2. HRQoL with all the participants. Statistically significant changes expressed with *) p < .05, and **) p < .005.

## Slide 3
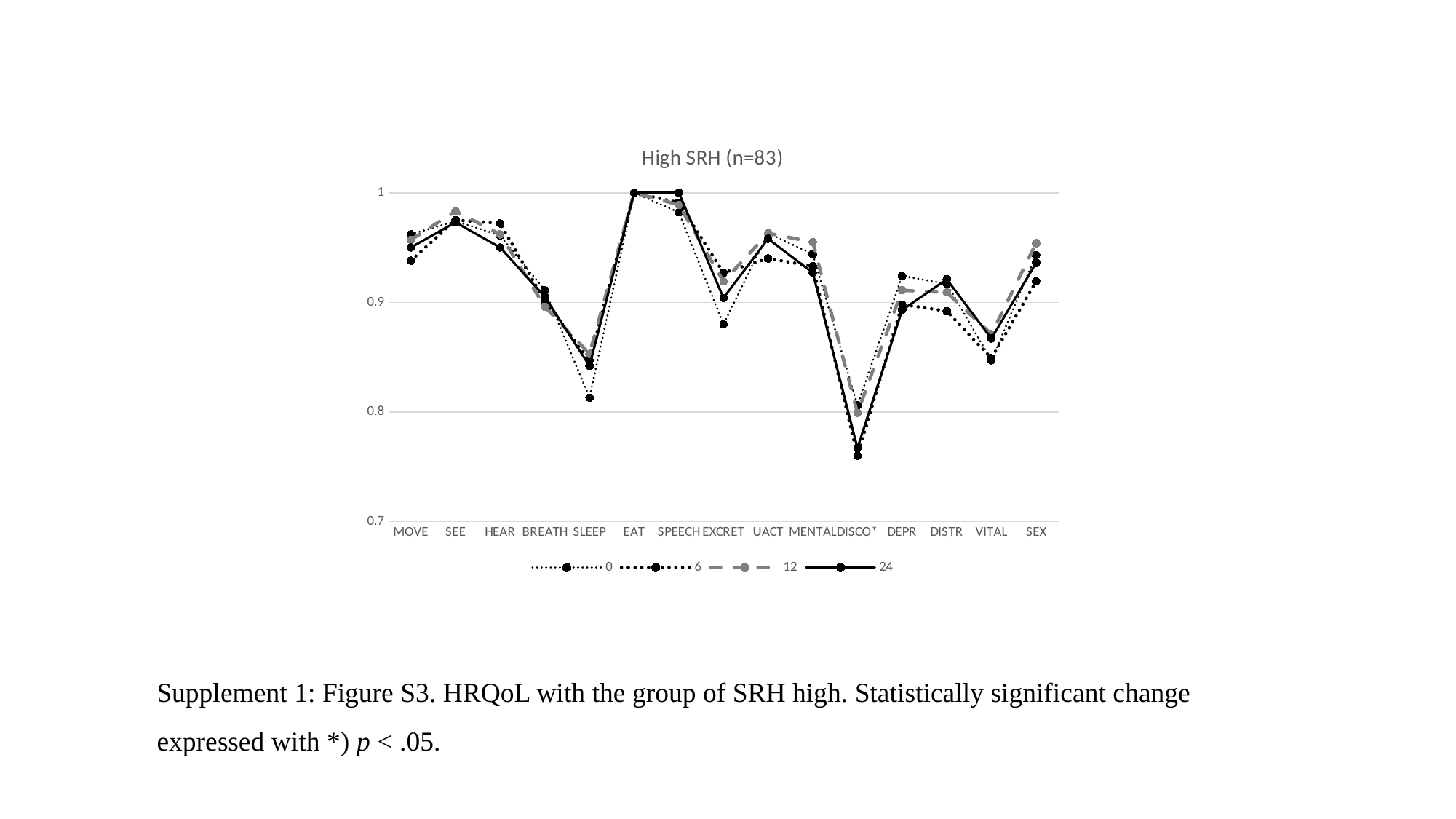

### Chart: High SRH (n=83)
| Category | 0 | 6 | 12 | 24 |
|---|---|---|---|---|
| MOVE | 0.962 | 0.938 | 0.957 | 0.95 |
| SEE | 0.974 | 0.975 | 0.983 | 0.973 |
| HEAR | 0.961 | 0.972 | 0.962 | 0.95 |
| BREATH | 0.911 | 0.901 | 0.896 | 0.905 |
| SLEEP | 0.813 | 0.847 | 0.853 | 0.842 |
| EAT | 1.0 | 1.0 | 1.0 | 1.0 |
| SPEECH | 0.982 | 0.991 | 0.989 | 1.0 |
| EXCRET | 0.88 | 0.927 | 0.919 | 0.904 |
| UACT | 0.963 | 0.94 | 0.963 | 0.958 |
| MENTAL | 0.944 | 0.933 | 0.955 | 0.927 |
| DISCO* | 0.806 | 0.76 | 0.799 | 0.767 |
| DEPR | 0.924 | 0.898 | 0.911 | 0.893 |
| DISTR | 0.917 | 0.892 | 0.909 | 0.921 |
| VITAL | 0.847 | 0.849 | 0.871 | 0.867 |
| SEX | 0.943 | 0.919 | 0.954 | 0.936 |Supplement 1: Figure S3. HRQoL with the group of SRH high. Statistically significant change expressed with *) p < .05.

## Slide 4
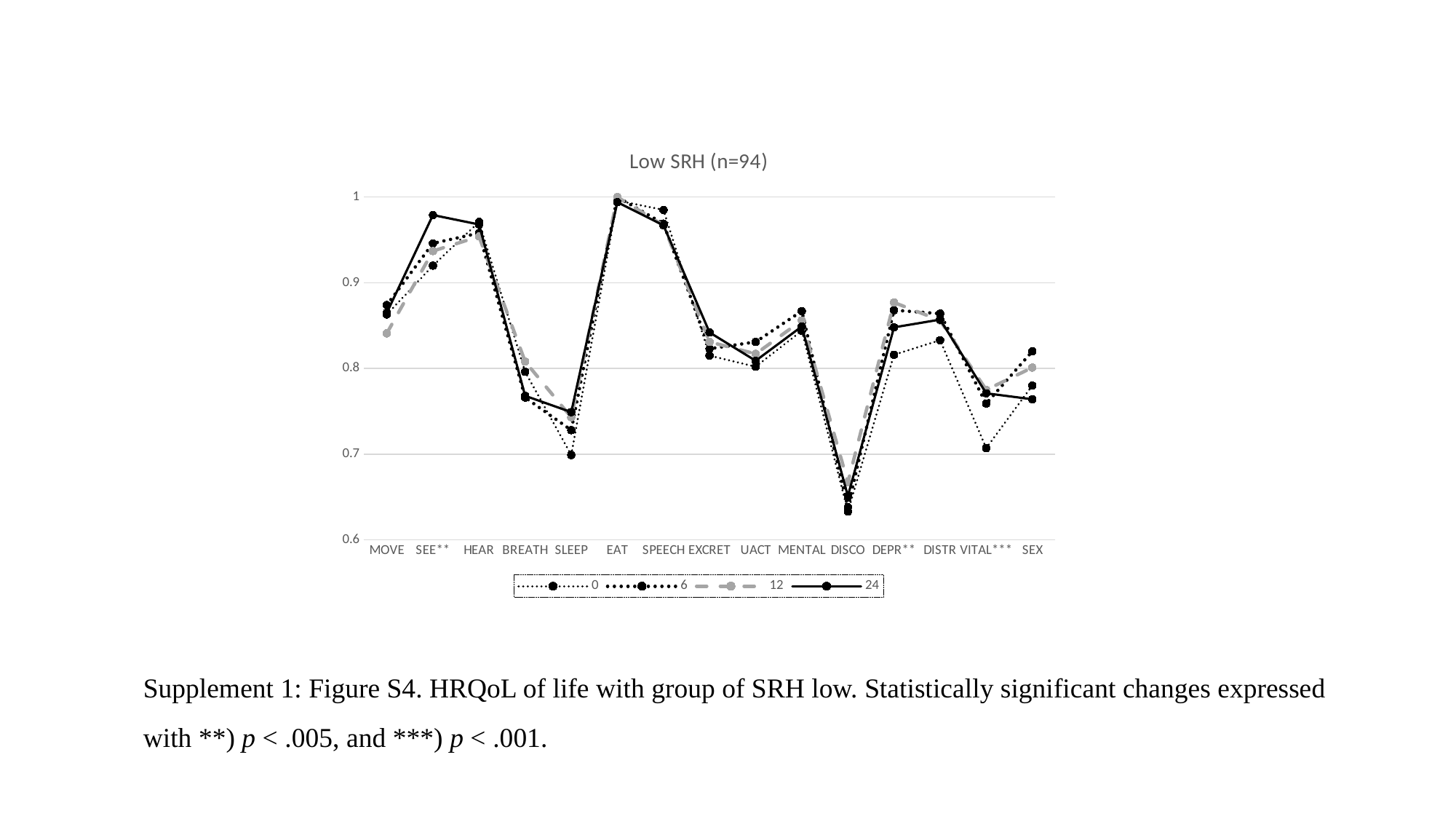

### Chart: Low SRH (n=94)
| Category | 0 | 6 | 12 | 24 |
|---|---|---|---|---|
| MOVE | 0.863 | 0.874 | 0.841 | 0.865 |
| SEE** | 0.92 | 0.946 | 0.937 | 0.979 |
| HEAR | 0.971 | 0.958 | 0.954 | 0.968 |
| BREATH | 0.796 | 0.766 | 0.808 | 0.768 |
| SLEEP | 0.699 | 0.728 | 0.743 | 0.749 |
| EAT | 0.996 | 1.0 | 1.0 | 0.994 |
| SPEECH | 0.985 | 0.969 | 0.967 | 0.967 |
| EXCRET | 0.815 | 0.823 | 0.831 | 0.842 |
| UACT | 0.802 | 0.831 | 0.817 | 0.809 |
| MENTAL | 0.844 | 0.867 | 0.856 | 0.849 |
| DISCO | 0.633 | 0.638 | 0.667 | 0.651 |
| DEPR** | 0.816 | 0.868 | 0.877 | 0.848 |
| DISTR | 0.833 | 0.864 | 0.856 | 0.857 |
| VITAL*** | 0.707 | 0.759 | 0.775 | 0.771 |
| SEX | 0.78 | 0.82 | 0.801 | 0.764 |Supplement 1: Figure S4. HRQoL of life with group of SRH low. Statistically significant changes expressed with **) p < .005, and ***) p < .001.
