# Supplementary material for: Changes in self-rated health, quality of life, and psychological flexibility among adults with overweight over a 24-month acceptance and commitment therapy–based lifestyle intervention
Source: Health Psychol Open. 2024 Nov 21;11:20551029241302977. doi: 10.1177/20551029241302977 (PMC11583280; doi:10.1177/20551029241302977)
Supplement: Supplemental Material - Changes in self-rated health, quality of life, and psychological flexibility among adults with overweight over a 24-month acceptance and commitment therapy–based lifestyle intervention [file sj-pdf-2-hpo-10.1177_20551029241302977.pdf]

Supplement 2: Table S1. Participant characteristics at baseline with the differences (*p*) between groups of SRH high and SRH low.

**Variable**	**All (*N* = 177)**	**SRH high (*n* = 83)**	**SRH low (*n* = 94)**	***p***
Sex, woman [b]	83.6% (*n* = 148)	85.5% (*n* = 71)	81.9% (*n* = 77)	.515
Age, mean (SD) [a]	52.81 (11.69)	51.59 (12.96)	53.88 (10.40)	.200
Working status employed [b]	49.2% (*n* = 87)	61.4% (*n* = 51)	38.3% (*n* = 36)	.002**
Participants having ≥1 long-term disease [b]	85.9% (*n* = 152)	75.9% (*n* = 63)	94.7% (*n* = 89)	< .001***
Weight, kg (SD) [a]	101.37 (21.01)	98.43 (20.28)	103.96 (21.41)	.081
BMI mean (SD) [a]	36.94 (6.74)	35.79 (6.40)	37.95 (6.90)	.033*
<30 [b]	13% (*n* = 23)	18.1% (*n* = 15)	8.5% (*n* = 8)	.059[c]
≥30 [b]	87% (*n* = 154)	81.9% (*n* = 68)	91.5% (*n* = 86)	.059[c]
Health-related quality of life, 15D mean (SD) [a]	0.87 (0.09)	0.92 (0.06)	0.83 (0.09)	< .001***
Psychological flexibility, mean (SD)				
AAQ-II [a]	16.31 (8.26)	13.06 (5.70)	19.18 (9.10)	< .001***
WBSI [a]	40.17 (13.24)	37.23 (12.59)	42.77 (13.32)	.005**
Self-rated health (SRH)				
Good	8.5% (*n* = 15)			
Quite good	38.4% (*n* = 68)			
Average	35% (*n* = 62)			
Quite poor	15.3% (*n* = 27)			
Poor	2.8% (*n* = 5)			

a = *t* test, b = Chi-Square Test, c = *p* value indicating the difference between the proportions of participants with BMI <30 and BMI ≥30 representing the groups SRH high and SRH low.

Supplement 2: Table S2. Means (variances) and statistically significant changes in total HRQoL and its dimensions (scale 0–1) among the whole sample, and among the two SRH groups over the 24-months intervention.

**Variable**	**Mean**	***p***	**Variance**	**Cohen *d***
ALL (N = 177)				
HRQoL, D15 Score				
W(3), 3.143, *p* = .370				
baseline	.874		.008	
change 0–6 months	.005	.276	.004	
change 6–12	.005	.391	.003	
change 12–24	-.006	.217	.003	
change 0–12	.010	.117		-.11
change 0–24	.004	.558		-.04
**Vitality**				
**W(3), 12.728, *p* = .005****				
baseline	.773		.031	
change 0–6	.030	**.049**	.035	
change 6–12	.016	.220	.022	
change 12–24	-.005	.742	.025	
change 0–12	.046	**.001**		**.27**
change 0–24	.041	**.006**		**.24**
**Sleeping**				
**W(3), 8.632, *p* = .035***				
baseline	.752		.043	
change 0–6	.032	**.032**	.035	
change 6–12	.008	.568	.024	
change 12–24	.000	.991	.032	
change 0–12	.041	**.014**		**.20**
change 0–24	.041	**.012**		**.21**
**Vision**				
**W(3), 8.836, *p* = .032***				
baseline	.946		.017	
change 0–6	.015	.111	.013	
change 6–12	.000	.962	.011	
change 12–24	.016	.103	.009	
change 0–12	.014	.218		.12
change 0–24	.030	**.004**		**.29**
SRH high (n = 83)				
HRQoL, D15 Score				
W(3), 1.281, *p* = .734				
baseline	.923		.003	
change 0–6 months	-.005	.429	.003	
change 6–12	.008	.389	.003	
change 12–24	-.006	.439	.002	
change 0–12	.002	.837		-.03
change 0–24	-.004	.729		.05
**Discomfort and symptoms**				
**W(3), 8.559, *p* = .036***				

baseline	.806		.025	
change 0–6	-.046	**.013**	.025	
change 6–12	.039	.075	.027	
change 12–24	-.033	.216	.029	
change 0–12	-.007	.784		.04
change 0–24	-.039	.155		**.22**
SRH low (n = 94)				
HRQoL, D15 Score				
W(3), 5.295, *p* = .151				
baseline	.831		.009	
change 0–6 months	.015	.035	.004	
change 6–12	.001	.874	.004	
change 12–24	-.007	.363	.003	
change 0–12	.016	.061		-.17
change 0–24	.010	.253		-.10
**Vitality**				
**W(3) 16.484, *p* = .001*****				
baseline	.707		.031	
change 0–6	.052	**.016**	.039	
change 6–12	.016	.366	.022	
change 12–24	-.005	.827	.025	
change 0–12	.069	**< .001**		**.40**
change 0–24	.064	**.001**		**.37**
**Depression**				
**W(3) 13.252, *p* = .004****				
baseline	.816		.027	
change 0–6	.052	**.002**	.023	
change 6–12	.009	.606	.022	
change 12–24	-.029	.155	.024	
change 0–12	.060	**.002**		**-.37**
change 0–24	.031	.144		**-.19**
**Vision**				
**W(3) 14.945, *p* = .002****				
baseline	.920		.026	
change 0–6	.026	.098	.021	
change 6–12	-.009	.603	.016	
change 12–24	.042	**.013**	.014	
change 0–12	.017	.388		**-.12**
change 0–24	.059	**< .001**		**-.48**

W = Wald test

Supplement 2: Table S3. Changes in psychological flexibility and thought suppression with the two groups of SRH over the 24-month intervention.

	**SRH high (*n* = 83)**				**SRH low (*n* = 94)**			
	Mean	*p*	Variance	Cohen *d*	Mean	*p*	Variance	Cohen *d*
**AAQ-II**	W(3), 1.985 *p* = .576				**W(3), 15.660, *p* = .001**			
baseline	13.06	< .001	32.08		19.18	< .001	81.98	
change 0 – 6 months	0.82	.261	35.06	-.12	-1.30	.085	46.51	.14
change 6–12	-1.18	.295	73.90	.15	**-1.75**	**.036**	47.34	**.19**
change 12–24	-0.01	.992	87.61	.00	0.70	.251	21.79	-.08
change 0–12	-0.36	.715		.05	**-3.05**	**< .001**		**.34**
change 0–24	-0.37	.722		.05	**-2.34**	**.004**		**.25**
**WBSI**	**W(3), 26.456, *p* < .001**				**W(3), 12.979, *p* = .005**			
baseline	37.23	< .001	156.51		42.77	< .001	175.56	
change 0 – 6 months	-0.72	.625	149.90	.05	-2.28	.073	118.48	.17
change 6–12	**-3.82**	**.018**	152.28	**.26**	-1.83	.104	69.34	.12
change 12–24	-1.52	.389	144.60	.11	0.45	.726	85.37	-.03
change 0–12	**-4.54**	**< .001**		**.33**	**-4.11**	**.002**		**.29**
change 0–24	**-6.06**	**< .001**		**.46**	**-3.66**	**.004**		**.26**

W = Wald test
